# 3-Year Real-World Outcomes with the Swedish Adjustable Gastric Band™ in France

**DOI:** 10.1007/s11695-012-0765-2

**Published:** 2012-09-30

**Authors:** G. Ribaric, J. N. Buchwald, G. d’Orsay, F. Daoud, for the French Health Technology Assessment Body (Haute Autorité de Santé [HAS]) Swedish Adjustable Gastric Band (SAGB™) Study Group

**Affiliations:** 1 Ethicon Endo-Surgery (Europe), European Surgical Institute, Hamburg, Germany; 2 Division of Scientific Writing, Medwrite Medical Communications, Maiden Rock, WI USA; 3 Johnson & Johnson Medical, Ethicon SAS, Paris, France; 4 Data Management & Biometrics, Medextens SARL, Paris, France; 5 Clinical and Medical Affairs, Ethicon Endo-Surgery (Europe) GmbH, MD&D EMEA (Europe, Middle East, Africa), Johnson & Johnson, Hummelsbütteler Steindamm 71, 22851 Norderstedt, Germany

**Keywords:** Laparoscopic adjustable gastric banding, Obesity, National outcomes, Swedish adjustable gastric band, SAGB, Quality of life, BAROS, EQ-5D, QALY

## Abstract

The study objective was to ascertain outcomes with the Swedish adjustable gastric band (SAGB) on an intention-to-treat basis in multiple centers across the French social health insurance system. SAGB results at 3-year follow-up are reported. The noncomparative, observational, prospective, consecutive cohort study design sought a 500-patient minimum recruitment geographically representative of continental France. Safety (adverse events [AEs], device-related morbidity, and mortality) and effectiveness (change in body mass index [BMI, kilograms per square meter], percentage excess weight loss, comorbidities, quality of life [QoL]) were assessed. Adjustable gastric band survival was calculated. Thirty-one surgeons in 28 multidisciplinary teams/sites enrolled patients between September 2, 2007 and April 30, 2008. SAGB was successfully implanted in 517 patients: 88.0 % female; mean age, 37.5 years; obesity duration, 15.3 years (baseline: mean BMI, 41.0; comorbidities, 773 in 74.3 % of patients; Bariatric Analysis and Reporting Outcome System (BAROS), 1.4; EuroQoL 5-Dimensions (EQ-5D), 0.61; EuroQoL–visual analog scale (EQ–VAS), 52.3). At 3 years: BMI, 32.2 (mean change, −9.0; *p* < 0.0001); excess weight loss, 47.4 %; comorbidities, 161 in 27.2 %; BAROS, 3.6 (+2.2, *p* < 0.0001); EQ-5D, 0.84 (+0.22, *p* < 0.0001); EQ–VAS, 73.4 (+21.4, *p* < 0.0001). SAGB-induced weight loss was associated with substantially improved QoL. One death occurred and was unrelated to the treatment. No AE was reported in 68.3 % of patients, and no confirmed device-related AE in 77.0 %. Overall AE rate was 0.19 per patient year. Device retention was 87.0 %. Analysis of patients lost to follow-up showed a nonsignificant effect on overall study results. In a prospective, consecutive cohort, “real-world”, nationwide study, the Swedish Adjustable Gastric Band was found safe and effective at 3-year follow-up.

## Introduction

Long-term effectiveness and safety of adjustable gastric banding (AGB) is of ongoing importance to patients, surgeons, and insurers. The most recent global survey of bariatric surgery, incorporating 36 respondent nations/national groupings, found that AGB utilization is in its ascendancy in the USA and Canada, having increased in this grouping 944.2 % between 2003 and 2008 (from 9,270 to 96,800 procedures annually). Even in Europe, where AGB use dropped 20.5 % relative to other bariatric procedures, the number of AGB operations per annum increased 34.2 % from 21,496 to 28,843 in the same time frame [1]. Selection of AGB as a bariatric treatment remains high and with several hundred thousand patients currently in long-term follow-up, intermediate and long-term national AGB outcome data are needed for insurance valuations and also as benchmarks for bariatric surgery outcome improvement.

Between 2003 and 2008, France performed the third greatest number of bariatric procedures annually (*n* = 13,722) after the USA and Brazil [1]. As estimated in 2007, the majority of bariatric procedures performed in France, 87.3 %, were AGBs [2]. As part of its responsibility to the social insurance system [3, 4], the French government commissioned safety and efficacy studies to obtain insurance data with which to evaluate the service to patients provided by the proprietary AGBs used in France (e.g., Swedish Adjustable Gastric Band™ [SAGB™]). The objective of the current study, requested by the *Commission d’Evaluation des Produits et Prestations* (a branch of the French Health Technology Assessment Body, Haute Autorité de Santé [HAS]) [5, 6] and sponsored by the manufacturer of the SAGB, Ethicon Endo-Surgery Europe, GmbH, was to prospectively ascertain outcomes typical of the French experience with the SAGB. The study protocol required recruitment of at least 500 consecutive SAGB patients (with <20 % attrition) by a representative sample of SAGB-implanting surgeons (i.e., both very experienced and less experienced) in urban and rural centers across regions of continental France [7]. We present SAGB cohort outcomes collected in 30 surgical sites between September 2, 2007 and November 20, 2011 analyzed on an intent-to-treat (ITT) basis with 3-year follow-up.

## Methods

### Study Protocol and Conduct

In 2007, at the request of the French HAS [5, 6], with the objective of data acquisition to assess reimbursement of the SAGB product in France, Ethicon Endo-Surgery Europe, GmbH undertook sponsorship of a nationwide study of SAGB outcomes in participating centers (registered in the Clinical Trials Web database, #NCT01183975 [7]). The sponsor developed the protocol and case report form to implement HAS requirements and good clinical practices (GCPs) defined in ISO EN 14155-1 and 2 [8, 9], with consideration for real-life study constraints. The protocol was approved by HAS, the Comité Consultatif sur le Traitement de l’Information en matière de Recherche dans le domaine de la Santé, and the Commission Nationale de l’Informatique et des Libertés (CNIL) to ensure patient welfare in the study methodology and the ethical conduct of the study. The study was monitored by Contract Research Organization, Medextens SARL, Paris, France.

All patients provided written informed consent prior to surgery in accord with GCP guidelines and the Declaration of Helsinki [10]. Treatment payments for patients were covered by French National Health Insurance [6]. An independent study-monitoring committee consisting of a medical nutritionist, a nonparticipating bariatric surgeon, and a pharmacologist audited study conduct and the interim report.

### Design, Setting, Recruitment

The study was of a prospective, multicenter, noncomparative observational design, with consecutive recruitment of SAGB patients by participating surgeons. Study objectives specified by HAS were the evaluation of (1) safety by occurrence of adverse events, (2) clinical effectiveness by weight reduction, and (3) comorbidity and quality of life (QoL) improvement. Beyond the protocol prescribing standardized safety and weight data collection and assessment, no other standardization of physical examinations, laboratory work, radiology studies, or follow-up procedure(s) (e.g., band adjustments) was feasible or appropriate, given the independent, real-life assessment of participating centers.

The study was designed to record safety and effectiveness in morbidly obese patients who underwent SAGB implantation in French hospitals. Patients were eligible for surgery when presenting with a body mass index (BMI, kilograms per square meter) ≥40 or ≥35 to <40 with comorbidities after failure of medical treatment in the absence of contraindications in accord with published guidelines [11, 12]. Patients in whom SAGB implementation was a bariatric re-intervention were also part of consecutive enrollment. A minimum recruitment goal of 500 patients with <20 % loss to follow-up at 3 years was targeted.

### Inclusion Criteria

Surgeons were selected in public, academic, and private institutions geographically distributed across continental France that had varying degrees of bariatric surgery volume. The goal was to include surgeons and patients who would provide SAGB outcomes representative of the broad French real-life practice. In compliance with GCP standards, all surgeons underwent training in the study protocol.

All patients consecutively undergoing SAGB implantation during the recruitment period by participating investigators were included in the cohort, providing that they resided in continental France and had signed informed consent. According to routine practice requirements, these patients were expected to meet French guidelines for bariatric surgery [5] (similar to National Institutes of Health [11] and the European Guidelines on Surgery of Severe Obesity [12]), and to be eligible based on the HAS criteria [5, 6].

### Variables

Primary safety variables were frequency of adverse events, mortality, and medical device-related morbidity. The primary effectiveness variable was ITT BMI change over the course of 3 years. Secondary effectiveness variables were absolute weight, excess weight, percentage excess weight loss (%EWL; i.e., difference in preoperative and follow-up weight divided by excess weight, calculated by Miller’s formulas [13–17] for ascertaining ideal weight, which correspond to the midpoint value of the medium-frame range on the Metropolitan Life Insurance Height and Weight Tables, multiplied by 100), health-related QoL, and proportional changes in comorbidities.

### QoL Instruments

The Bariatric Analysis and Reporting Outcome System (BAROS), introduced by Oria and Moorehead in 1998 [18, 19], is a well-validated bariatric-specific QoL assessment that integrates weight loss data, comorbidity changes, and subjective QoL, complications, and reoperations. A maximum of three points are recorded within domains of weight loss, comorbidity improvement, and subjective QoL; points are deducted for complications and reoperations. The final score classifies outcomes as failure (≤1), fair (>1 to 3), good (>3 to 5), very good (>5 to 7), or excellent (>7 to 9).

The EuroQoL 5-Dimensions (EQ-5D) was selected as the generic complement to the BAROS due to its usefulness in calculating quality adjusted life years (QALYs). Specifically, the EQ-5D has proven effective in the estimation of the relative cost effectiveness associated with obesity interventions, including AGB [20]. The EQ-5D is a generic health-related QoL assessment comprised of five items and a EuroQoL–visual analog scale (EQ–VAS) [21–24] yielding a patient health profile along five dimensions mobility, self-care, usual activities, pain/discomfort, and anxiety/depression. Each dimension is represented by one item with three response options no problem, some problems, and severe problems. Responses to these five items can be normatively weighted to derive an EQ-5D utility score with a range of −0.594 to 1 (1 = ultimate health). A difference of ≥0.07 in EQ-5D utility has been identified as clinically important [24]. The EQ–VAS component represents a single-item global QoL assessment in which patients are asked to rate their current health on a scale from 0 (worst imaginable) to 100 (best imaginable) [25].

### Technique, Band Adjustment

Gastric banding was performed via standardized pars flaccida technique [26]. SAGB model options were 2100-X (with locking ring and injection port); 2200-X Quick-Close; and BD2XV Quick-Close with Velocity™ injection port. Band adjustments were performed according to the discretion of participating surgeons.

### Data Collection

Baseline patient characteristics were collected (e.g., gender and age); weight, obesity-related comorbid disease, and QoL were recorded preoperatively, and assessed at 1, 3, 6, 12, 18, 24, and 36 months postoperatively. Comorbidity data were obtained via questionnaire with diagnoses established according to each investigator’s usual practice.

Remote entry of clinical data was performed by investigators or administrative support personnel via password-protected access to the Medextens-Medalliance eCRF Manager (v.1.3) web database (CNIL registered, www.medalliance.fr). Patients were informed of their rights of access, correction, and refusal to participate.

### Statistical Analysis

Stata/MP software (v.11.2, StataCorp LP, College Station, TX, USA) was used to perform all statistical analyses. Statistical command syntax was programmed as a repeatable and auditable script. Statistical analysis was performed according to International Conference on Harmonization E9 guidance. No statistical justification for sample size is presented herein, as determination of the number of consecutive patients required was pre-established by HAS specifications. Continuous demographic variables were reported as mean, standard deviation (SD), and 80 % interpercentile range. Categorical demographic variables (including preoperative BMI subgroups) were reported as number and percentage. Comorbidity data and adverse events were also reported as number and percentage. Implant-survival rate and associated 95 % confidence interval (CI) was calculated using the Kaplan–Meier method. Continuous outcome variables were generally reported as mean, SD, and 95 % CI. A qualitative assessment of comorbidities before and after SAGB implantation was carried out. Fisher’s exact test was used to investigate relationships between categorical variables. Between-group comparisons along continuous measures were conducted using two-tailed, independent-sample *t* tests. In addition, a series of two-tailed, single-sample *t* tests were performed to assess within-patient change from baseline, adopting a null hypothesis of no significant mean change in weight and QoL variables (*H*_0_ = 0, based on the nature of the disease of obesity without intervention). The Shapiro–Wilk test for normality was conducted on each variable distribution prior to *t* test. Linear regression was applied in the analysis of relationships between QoL measures, as well as in the relationship between BMI and QoL. Statistical significance was set at *p* ≤ 0.05.

## Results

Initially, 51 surgeons were identified as trained to implant the SAGB based on previous year records of performing ≥1 bariatric procedure weekly and, specifically, 35 AGB procedures annually. Forty surgeons enlisted, 37 created patient records in the database, 31 of which actively contributed to the cohort (note: five surgeons reconsidered their ability to effectively participate in the study and voluntarily withdrew during the patient recruitment phase; one surgeon was excluded from the study during the data validation process due to marked protocol noncompliance; the 63 patient records created by the six surgeons were deemed invalid). These 31 surgeons were part of 28 multidisciplinary teams in five university hospitals, three general hospitals, and 23 private institutions. Patient screening and enrollment began September 2, 2007 and concluded April 30, 2008. The final 3-year follow-up visit was due on April 30, 2011 but was extended to November 20, 2011 to accommodate patient availability.

The 31 surgeons who contributed to the cohort performed a total of 1,239 bariatric procedures during the study period, of which 560 cases were recorded in the online database as consecutive SAGB patient records. Among the 560 records, 43 were deemed invalid because they contained data entry errors, were duplicates, SAGB implantation was not documented, or because patients withdrew. Overall, 517 valid consecutive patients records were included in the database analysis. Participating surgeons performed an average of 16.7 SAGB implants. The majority of cases (514/517, 99.4 %) were performed laparoscopically; conversions to laparotomy were reported in two cases (0.39 %); 99.6 % (515/517) used pars flaccida technique; 76.4 % (395/517) employed band fixation; and 49.13 % (254/517) used port fixation. Immediate band filling occurred in just 0.58 % (3/517) of cases. Overall, 66.54 % (344/517) of SAGB implants were performed using model 2200X; model BD2XV was used in 33.27 % (172/517) of cases.

Safety and weight data met completeness and accuracy criteria required by the protocol. Three-year clinical outcome data were available in 85.9 % (444/517) of the cohort; the objective that <20 % of patients lost to follow-up at 3 years was reached. Due to the observational nature of this study with significant differences between surgeons in terms of their routine practice, comorbidity and QoL data did not meet protocol-targeted completeness; comorbidity data was available for 78.0 % of the SAGB cohort at 3-year follow-up, while QoL data availability at 3 years ranged from 39.0 % to 78.0 %, depending on the complexity of the instrument.

### Baseline Patient Characteristics

In the 517 consecutive patients with confirmed SAGB implants, gender and age were reported in 516 patients (99.8 %); disease duration in 504 patients (97.5 %); height, weight, excess weight, and BMI in 513 patients (99.2 %). The cohort consisted of 88.0 % (*n* = 455) female and 11.8 % (*n* = 61) male, with a mean age of 37.5 ± 10.9 years, obesity duration of 15.3 ± 8.4 years, BMI of 41.0 ± 4.9 kg/m^2^, absolute weight of 111.4 ± 16.8 kg, and excess body weight of 52.1 ± 14.1 (Table 1).

**Table 1:** Baseline characteristics for 517 total patients receiving SAGB implant

Characteristic	Value
Gender	
Male, *N* (%)	61 (11.8)
Female, *N* (%)	455 (88.01)
Age, mean ± SD, years (80 % IPR)	37.5 ± 10.9 (23.4 to 53.7)
Duration of obesity, mean ± SD, years (80 % IPR)	15.3 ± 8.4 (6.0 to 28.0)
Height, mean ± SD, m (80 % IPR)	1.7 ± 0.1 (1.6 to 1.8)
Absolute weight, mean ± SD, kg (80 % IPR)	111.4 ± 16.8 (93.0 to 133.0)
Excess body weight, mean ± SD, kg (80 % IPR)	52.1 ± 14.1 (37.8 to 71.0)
BMI, mean ± SD, kg/m^2^ (80 % IPR)	41.0 ± 4.9 (36.1 to 47.3)
BMI ≥40, kg/m^2^, *N* (%)	291 (56.3)
BMI ≥35 and < 40, kg/m^2^, *N* (%)	193 (37.3)
BMI <35, kg/m^2^, *N* (%)	29 (5.6)
At least 1 comorbidity, *N* (%)	384 (74.3)
History of family obesity, *N* (%)	343 (66.3)
1-year specialized medical care and multidisciplinary assessment, *N* (%)	475 (91.9)
First bariatric intervention, *N* (%)	471 (91.1)
Preoperative psychological evaluation, *N* (%)	503 (97.3)
BAROS^a^, mean ± SD (80 % IPR)	1.4 ± 1.4 (−0.3 to 3.0)
EQ-5D^b^, mean ± SD (80 % IPR)	0.61 ± 0.31 (0.09 to 0.88)
EQ–VAS^c^, mean ± SD (80 % IPR)	52.3 ± 18.4 (30.0 to 80.0)

*SD* standard deviation, *IPR* interpercentile range (the 80th IPR indicates variable values ranging from 10th to 90th percentile), *BMI* body mass index, *BAROS* Bariatric Analysis and Reporting Outcome System, *EQ-5D* EuroQoL 5-Dimensions, EQ–VAS EuroQoL-Visual Analog Scale

^a^ *N* = 302 for BAROS data recorded at 30 days postoperative

^b^ *N* = 449 with EQ-5D data at inclusion

^c^ *N* = 446 with EQ–VAS data at inclusion

AGB was the first bariatric surgery in 471 patients (91.1 %) and a re-intervention in 43 patients (8.3 %). There were 291 patients (56.3 %) with a BMI of ≥40; 193 (37.3 %) with a BMI of ≥35 to <40; 29 patients (5.6 %) presented with a BMI of <35. Baseline BMI could not be calculated in four patients (0.77 %) due to missing weight and/or height data.

Patients with ≥1 comorbidity numbered 384 (74.3 %), with a total of 781 recorded comorbidities. A history of family obesity was reported in 343 patients (66.3 %) and 475 patients (91.9 %) had ≥1 year of specialized medical care prior to SAGB. Mean BAROS was 1.4 ± 1.4, mean EQ-5D was 0.61 ± 0.31, and mean VAS was 52.3 ± 18.4.

### Adverse Events

**Fig. 1:**
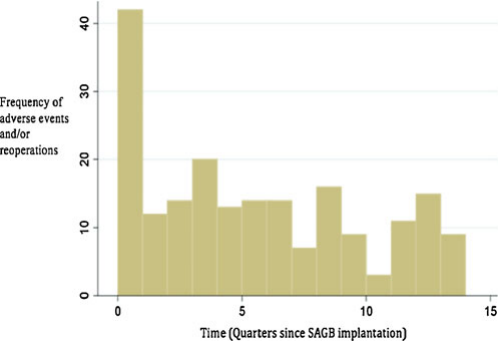
Time distribution of adverse events or re-interventions

The majority of SAGB patients (68.3 %, 353/517) experienced no adverse events (AEs) of any kind. Also, no confirmed device-related AE or serious adverse events (SAEs; life-threatening or disabling event requiring intervention) was reported in 77.0 % (398/517). Confirmed AEs and/or SAEs associated with the SAGB procedure totaled 290 in 164 patients (31.7 %) intraoperatively and up to 3-year follow-up. There were 153 (29.6 %) AEs and 137 (26.5 %) SAEs constituting an overall rate of 0.19 adverse event per patient year. There was one death caused by an accident unrelated to the procedure. Figure 1 shows the distribution of the 290 confirmed events with a peak event frequency of 42 events during the first quarter following SAGB implant; thereafter, frequency was approximately 10 events per quarter.

Among 290 confirmed events, there were 143 device-related events in 119 patients (23.0 %); thus, the device-related event rate was 0.09 event per patient year. The most frequent device-related AEs were 22 (4.3 %) band slippages, 14 (2.7 %) esophageal dilations, 13 (2.5 %) pouch dilations, 13 (2.5 %) port malpositions, 9 (1.7 %) port rotations, and 7 (1.4 %) port disconnections, 5 (1.0 %) food intolerances, 5 (1.0 %) dysphagia, 4 (0.8 %) band infections, 3 (0.6 %) port infections, 3 (0.6 %) band-related blockages, and 3 (0.6 %) band-related records of frequent vomiting. Device-related SAEs included 67 (13.0 %) band removals, 23 (4.4 %) abdominoplasties, 22 (4.3 %) port re-interventions without port removal, 8 (1.6 %) port removals, and 3 (0.6 %) band re-interventions without removal.

### Implant Survival Rate Function

**Fig. 2:**
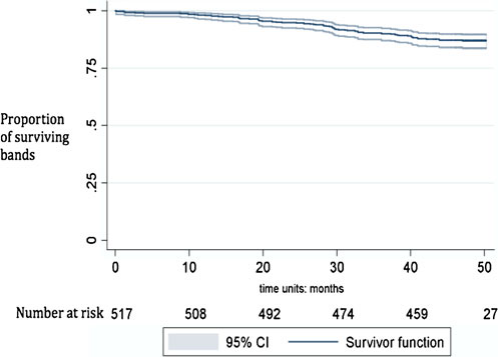
Kaplan–Meier band survival curve

SAGB removals occurred at different intervals post implantation. The risk of failure (SAGB removal) versus the chances of success (SAGB retention) over time was estimated with Kaplan–Meier cumulative survival function. All reported removals were included in the analysis (Fig. 2). Patients with SAGB in place at the date of database freeze (November 20, 2011) were censored. The 517 valid implanted patients were exposed to a time-at-risk of 23,386 months. Sixty-seven failures were confirmed; band removal was typically associated with the most severe adverse events. Median time at risk was 48 months and the last recorded failure date was 50.23 months following implantation. At 3 years, SAGB implant survival rate was 87.0 % (95 % CI: 83.7, 89.6).

### Weight Loss

**Fig. 3:**
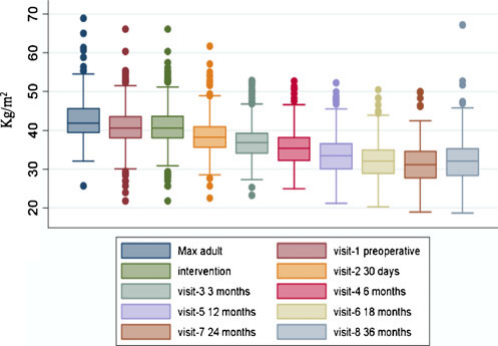
SAGB cohort body mass index evolution

Three-year postoperative outcomes were available in 85.9 % (444/517) of patients. Intention-to-treat BMI change over the course of 3 years was available in 423 (81.8 %) patients, including 373 patients with SAGBs still in place and 50 patients whose bands were removed. BMI change could not be calculated in 94 patients, including 73 lost to follow-up with unknown outcomes, 13 with band removals, 7 with incomplete data, and 1 death. Mean absolute weight was 87.1 ± 17.6 kg (85.4, 88.8) compared to 111.6 ± 16.9 kg (110.0, 113.2) at baseline (Table 2). This represented a mean absolute weight reduction of 24.5 ± 14.6 kg (23.1, 25.9; *t*[422] = 34.4, *p* < 0.0001). Mean excess body weight was 28.0 ± 15.8 kg (26.5, 29.5) compared to 52.5 ± 14.3 kg (51.1, 53.8) at baseline, a reduction in excess weight of 24.5 ± 14.7 kg (23.1, 25.9; *t*[421] = 34.3, *p* < 0.0001) corresponding to a %EWL of 47.4 ± 32.1 (44.3, 51.0). Mean BMI was 32.2 ± 5.8 (31.6, 32.7), down approximately 22.0 % from 41.2 ± 5.0 (40.7, 41.7). This change in patient BMI represented a mean reduction of 9.0 ± 5.3 (8.5, 9.5; *t*[422] = 35.0, *p* < 0.0001). Figure 3 depicts the cohort’s evolution in mean BMI, beginning with a maximum adult BMI of 42.8 ± 4.9 (42.3, 43.2).

**Table 2:** Three-year weight outcomes and mean change assessment

Weight variable	Value			*P* value^b^
	Mean ± SD (95 % CI)			
	*N* = 423^a^			
	Preoperative	3 Years	Mean change	
Absolute weight, kg	111.6 ± 16.9 (110.0, 113.2)	87.1 *±* 17.6 (85.4, 88.8)	−24.5 ± 14.6 (−25.9, −23.1)	<0.0001
Excess body weight, kg	52.5 ± 14.3 (51.1, 53.8)	28.0 ± 15.8 (26.5, 29.5)	−24.5 ± 14.7 (−25.9, −23.1)	<0.0001
BMI, kg/m^2^	41.2 ± 5.0 (40.7, 41.7)	32.2 ± 5.8 (31.6, 32.7)	−9.0 ± 5.3 (−9.5, −8.5)	<0.0001
% EWL	–	47.4 ± 32.1 (44.3, 51.0)	–	–

*BMI* body mass index, *EWL* excess weight loss, *SD* standard deviation, *CI* confidence interval

^a^ *N* = 423 for absolute weight and BMI within-patient change calculations; *N* = 422 for excess weight within-patient change and %EWL calculations

^b^ *P* values obtained from single-sample two-tailed *t* tests assessing mean weight change in patients with complete preoperative and 3-year follow-up data

A significant difference in preoperative mean BMI was observed between patients receiving SAGB as a first intervention and those receiving SAGB as a re-intervention (41.4 ± 4.4 vs 37.1 ± 7.4, *p* < 0.001); however, by 3-year follow-up, mean BMI was not significantly different (32.1 ± 5.8 vs 32.8 ± 5.6; *p* = 0.51) between these two groups of patients. In order to assess the risk of bias in estimating the primary endpoint on an ITT basis, patients with and without 3-year follow-up weight data were compared on several variables that could potentially influence BMI change. Mean preoperative BMI of patients with complete 3-year weight data relative to those without were not significantly different (41.2 ± 5.0 vs 40.3 ± 4.6; *p* = 0.10). Age at baseline, gender, duration of disease, maximum adult BMI, SAGB as first intervention or re-intervention, SAGB model type, and frequency of band removal were also not significantly different between groups.

In addition, the magnitude of the effect of including patients with band removal in the calculation of BMI change over 3 years was also assessed. As expected, a significant difference in mean BMI change was observed between patients with band ablation vs patients with intact SAGBs at 3 years (−6.2 ± 5.5 vs −9.4 ± 5.2, *p* < 0.001); however, the inclusion of patients with band ablation (in conformance with ITT analysis) had a nonsignificant effect on the overall cohort mean change value (−9.0 ± 5.3 [−9.7, −8.4] vs −9.4 ± 5.2 [−10.1, −8.7]). Overall, BMI reduction was observed across visits with most of the reduction occurring during the first postoperative year, continuing through the second year, followed by a negligible BMI increase during the third year (Fig. 3).

### Comorbidities

Adhering to the study’s observational design, the protocol did not seek to modify current diagnostic or therapeutic practices: no protocol-driven diagnostic tests were required. At each follow-up visit, comorbidities were generally reported as “present” or “absent.” Significant variation in diagnostic methodology, terminology, and reporting regularity was noted. Investigators, themselves, often did not assess comorbidities, but rather, reported assessment results provided to them by third parties. As a result, it was determined that an underreporting bias was a distinct possibility, thus limiting the reliability of a quantitative estimate of change in prevalence.

Arthropathy (38.0 %), hypertension (22.0 %), dyslipidemia (20.0 %), gastroesophageal reflux disease (20.0 %), obstructive sleep apnea (12.0 %), and diabetes (10.0 %) were the most frequently cited comorbidities at baseline, each experienced by at least 10 % of the cohort. Qualitative analysis suggested a continued reduction in the overall number of comorbidites over time and a gradual increase in number of patients with no reported comorbidities. Using recorded weight as an indicator of data integrity, 773 comorbidties were reported at baseline in 74.3 % (381/513) of patients with concomitant weight data, while no comorbidity was reported in 25.7 % (132/513). At 3-year follow-up, 161 comorbidities were reported in 27.2 % (116/426) of patients with concomitant weight data, while no comorbidity was reported in 72.8 % (310/426). In addition, the number of patients with multiple comorbid conditions was reduced at 3 years and the mean number of comorbidities per patient fell from 1.5 at baseline to 0.4 at 3-year follow-up. Analysis also suggested a slight increase in the average number of comorbidities per patient from year  2  (visit 7) to year  3  (visit 8), paralleling the modest weight regain over the same time period.

### Quality of Life

**Fig. 4:**
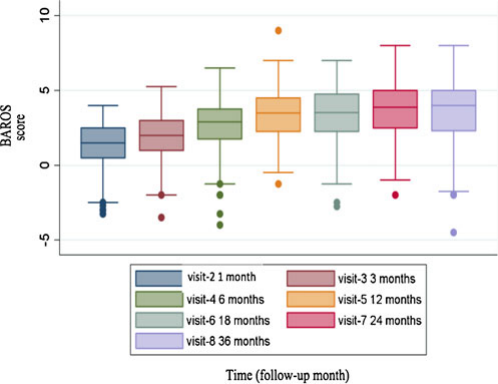
Evolution of SAGB cohort quality of life scores as measured by the Bariatric Analysis and Reporting Outcome System (BAROS®)

Although patient QoL assessment participation did not meet the protocol objective for data completeness, there were sufficient data to carry out quantitative analyses. [Note: Patient participation diminished with the complexity of the three QoL instruments.] All measures of QoL over time were significantly improved (Table 3). At 3 years, for patients with complete data, mean BAROS was 3.6 ± 2.2 (3.2, 4.0) compared to 1.4 ± 1.3 (1.2, 1.6) at pseudo-baseline (i.e., 30 days post SAGB). This positive change in bariatric-specific QoL represented a mean increase of 2.2 ± 2.2 (1.8, 2.6; *t*[133] = 11.9; *p* < 0.0001). Figure 4 depicts the cohort’s upward trend in mean QoL as assessed by BAROS over the course of the study. Mean EQ-5D utility score was 0.84 ± 0.21 (0.82, 0.86) relative to a baseline value of 0.62 ± 0.31 (0.59, 0.65). This represented a mean within-patient QoL improvement of 0.22 ± 0.32 (0.19, 0.25; *t*[346] = 12.6; *p* < 0.0001), thus, a mean utility gain of 0.66 QALY over 3 years. Mean EQ–VAS score was 73.4 ± 17.1 (71.6, 75.2) compared to 52.0 ± 18.4 (50.1, 53.9), a QoL increase of 21.4 ± 22.8 (19.0, 23.7; *t*[353] = 17.7; *p* < 0.0001).

**Table 3:** Three-year quality of life outcomes and mean change assessment

QoL variable	Value			*P* value^b^
	Mean ± SD (95 % CI)			
	Baseline	3 Years	Mean change	
BAROS	1.4 ± 1.3^a^ (1.2, 1.6)	3.6 ± 2.2 (3.2, 4.0)	2.2 ± 2.2 (1.8, 2.6)	<0.0001
EQ-5D	0.62 ± 0.31 (0.59, 0.65)	0.84 ± 0.21 (0.82, 0.86)	0.22 ± 0.32 (0.19, 0.25)	<0.0001
EQ–VAS	52.0 ± 18.4 (50.1, 53.9)	73.4 ± 17.1 (71.6, 75.2)	21.4 ± 22.8 (19.0, 23.7)	<0.0001

*BAROS* Bariatric Analysis and Reporting Outcome System, *EQ-5D* EuroQoL 5-Dimensions, *EQ*–*VAS* EuroQoL–visual analog scale, *SD* standard deviation, *CI* confidence interval

^a^BAROS data at 30 days postoperative functions as pseudo-baseline

^b^ *P* values obtained from single-sample two-tailed *t* tests assessing mean QoL change in patients with complete preoperative and 3-year follow-up data (i.e., *N* = 134 for BAROS, *N* = 347 for EQ-5D, *N* = 354 for EQ–VAS)

**Fig. 5:**
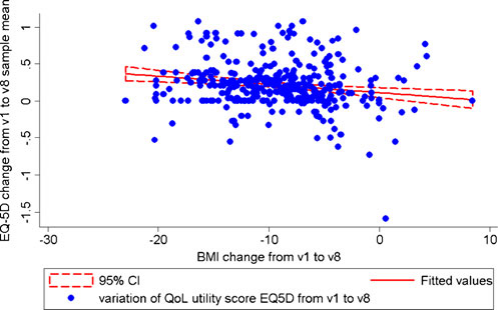
Relationship between body mass index reduction and quality of life changes as measured by EuroQoL 5-dimensions (EQ-5D). v1 = baseline; v8 = 3-year follow-up

Regression analysis indicated a significant association between weight loss and the EQ-5D (although weight per se is not a focus of the EQ-5D questionnaire). Using EQ-5D individual change scores as the response variable while controlling for baseline BMI, BMI reduction over the 3-year period following SAGB intervention was significantly related to an increase in global QoL (adjusted *R* ^2^ = 0.03; *F*(2, 339) = 6.93; *p* < 0.01; Fig. 5). In addition, individual change scores on the BAROS and the EQ-5D were shown to correlate at *r* = 0.43, *p* < 0.01. Overall QoL results suggest that EQ-5D, a patient-reported QoL outcome measure with minimal response burden, may be a sufficient measure of QoL following SAGB surgery.

## Discussion

The current study was representative of the SAGB standard of care in France and met the objectives defined in the protocol in line with CEPP requirements. Results were supportive of the clinical safety and effectiveness of the SAGB, as concluded in prior studies. Clinical outcomes were available for 86 % of the SAGB cohort; analysis indicated that patient data lost to follow-up over 3 years had no significant effect on treatment outcomes.

No treatment-related mortality was reported. AGB operative mortality is typically <0.1 % [27, 28] and 30-day to 2-year mortality, 0.07 % [29]. Risk related to this treatment consisted of adverse events or SAEs requiring intervention in 31.7 % of patients, including device-related events requiring intervention in 23.0 %. Band removal occurred in just 13.0 % of patients and was usually associated with the most severe adverse events or lack of procedure effectiveness. At 3-year follow-up, device retention was 87.0 %. Analysis suggested a reduction in overall comorbidites and an increase in the number of patients with no reported comorbidity.

A significant and sustained BMI reduction of 9.0 was observed, corresponding to 47.4 % EWL. BMI reduction was significantly associated with an increase in patient QoL, as has been shown in other AGB studies [30]; the BAROS scale showed a significant 2.2-point improvement from 30-day visit to 3-year follow-up. On average, SAGB patients tended to move from a BAROS QoL rating of “fair” to “good.” Also, SAGB patient EQ-5D Qol-related utility score significantly improved by 0.22 points (three times the reported minimally important difference) representing a utility gain of 0.66 QALY. In addition, the SAGB cohort’s EQ-5D mean utility score of 0.84 ± 0.21 at 3 years was slightly higher than that recorded for a UK general practice reference group of normal BMI patients (*n* = 782), EQ-5D = 0.80 ± 0.22 [31]. Overall, patient QoL change scores on BAROS and EQ-5D were shown to be significantly correlated (*r* = 0.43, *p* < 0.01) and both instruments were sensitive to BMI reduction over time. Results suggested that the EQ-5D, a self-report generic QoL measure with minimal response burden, may be a sufficient measure of QoL change following SAGB surgery. Finally, at 3-year follow-up, trend analysis indicated a modest and nonsignificant weight increase (*p* = 0.14) beginning after year  2 . Also, a corresponding decrease in QoL and a related increase in the average number of comorbidities suggested that SAGB patients may require more support after year  2 .

As of April 25, 2012, more than 2,300 peer-reviewed articles addressed the topic of AGB treatment [32]. The preponderance of these studies are of an observational design, in addition to several dozen randomized controlled trials (RCTs), most conducted in bariatric surgery “centers of excellence” (COEs)—experienced, high-volume medical centers that incorporate a multidisciplinary team approach [33] and compulsory pre- and post-operative protocols conforming to professional [34–36] and national [5, 11, 12] guidelines. Outcomes achieved in COE-standard sites may not be comparable to those achieved in less-experienced, lower-volume sites. Yet, non-COE sites represent a sizeable number of centers performing bariatric surgery, a fact important to insurers assessing the value of AGB treatment on a national scale. Thus, while seven high-volume single-center AGB studies over the last 10 years reported ≥55.0 % EWL at ≥3 years [37–43], integrated COE- and non-COE nationwide %EWL results are somewhat lower [44, 45].

The current study’s findings should be discussed in the context of evidence from other national AGB studies with intermediate-term follow-up, yet such reports are few. In the last decade, fewer than three dozen national bariatric surgery studies (16 large sample, retrospective, database analyses [46–61], 14 prospective surveys [1, 2, 62–73], and one ongoing [multi-article], nonrandomized, prospective controlled study [74]) have been published in the peer-reviewed literature, 14 of these featuring European countries. In the seven nationwide studies with ≥2-year follow-up data in morbidly obese patients (five observational AGB or AGB-inclusive studies and two French AGB surveys [2, 26, 58, 71, 74–76]), weight loss was less than or comparable to that of the current SAGB cohort.

In 2003, Angrisani et al. published the Italian Lap-Band experience with 1,863 patients in 27 centers, one of the earliest and largest nationwide AGB studies with intermediate-term follow-up based on a retrospectively analyzed national database, the Italian Group for Lap-Band (GILB) [58]. At baseline, patients had a mean BMI of 43.7 and 34.1 at 3 years post-AGB treatment, a reduction of 9.6 with >70 % follow-up (%EWL was not reported). These results are similar to those of the current French SAGB cohort where baseline BMI was 41.0, and 32.2 at 3 years, a reduction of 9.0 (*p* < 0.0001) with 81.8 % follow-up. In 2012, a subsequent nationwide AGB study of the same GILB database, a comparison of the perigastric (PG) and pars flaccida (PF) techniques was undertaken by Di Lorenzo et al. [26] in 2,549 Lap-Band patients (baseline BMI, 46.4): 1,206 (47.3 %) were operated via PG, 1,343 (52.7 %) via PF, approach. In patients eligible for minimum 3-year follow-up, respective mean BMI was 33.8 (a reduction of 12.6) and 32.4 (−14.0), and mean EWL was 47.2 % and 48.9 %. These results, as in the earlier GILB cohort [58] are essentially equivalent to those of the current study.

The third nationwide AGB report, by Phillips et al., a prospective US Food and Drug Administration trial of the SAGB (the “Realize® Band” in the USA) was conducted in 12 academic and private centers with surgeons versed in laparoscopic surgery but with varied AGB experience [75]. Of 405 patients screened, 276 qualified for the study and underwent band surgery. Mean baseline BMI was 44.5 with a reduction of 8.2 at 3 years and 83.0 % follow-up; EWL in the REALIZE trial was 41.1 % compared to 47.4 % in the current French SAGB cohort.

Two nationwide AGB-related government-commissioned surveys have been conducted in France. Chevallier et al. [71] published a prospective, consecutive series, statistical analysis of factors predictive of AGB outcomes from a national public insurance perspective. Although this survey lacked 3-year data, it found that EWL was <50.0 % at 1–2 years in the majority of 1,079 morbidly obese adults who had undergone the AGB procedure [71]. Basdevant et al. [2] studied current bariatric surgery practices across procedures in the same French National Medical Insurance Service registry as that studied by Chevallier et al. They showed a BMI reduction of 9.9 and EWL of 46.0 % at 2 years, roughly equivalent to the French SAGB cohort 3-year outcomes. The single prior French clinical trial of an AGB with 3-year follow-up, a study of the MIDBand (MID, Dardilly, France), was conducted in 13 centers (two academic, one public general hospital, 10 private clinics) in 262 morbidly obese adults, 193 of whom were included in the weight-loss analysis (26 % lost to follow-up). Mean baseline BMI was 41.8 with a reduction of 8.2–30.7 at 3 years; median EWL was 61.0 % [76]. Percentage EWL ranges reported at 2–3 years in the three prior French AGB studies, taken together with the French SAGB cohort study, are likely representative of current AGB weight loss outcomes in France.

Chevallier et al. also found that, independent of patient characteristics, the key predictor of AGB success was surgical volume (i.e., multidisciplinary team typically performing >2 bariatric procedures per week) [71]. Interestingly, although the current French SAGB study incorporated centers that performed <2 bariatric procedures per week, its mean %EWL exceeded or equaled that of five of the six other national AGB studies summarized.

A final prominent, long-running, nationwide clinical trial, the Swedish Obese Subjects (SOS) study, also reported BMI reduction less than that of the current 3-year French study, although it is typical for weight loss to diminish somewhat after reaching its peak between 1 and 3 years. The SOS study is an ongoing, nonrandomized, prospective, controlled study that has followed 480 primary healthcare sites and 25 public surgical departments in Sweden since the recruitment phase (September 1, 1987 to January 31, 2001). Bariatric surgery (gastric bypass, vertical banded gastroplasty, or AGB [*n* = 156 at baseline]) was performed on 2,010 morbidly obese patients and 2,037 contemporaneously matched morbidly obese controls received usual care in the Swedish primary care system. In 2004 (10-year follow-up), the reported mean BMI in the AGB subgroup (*n* = 156) had decreased by 5.4 (12.8 %, *p* < 0.05) to 36.5 from 41.9 ± 4.2 (total surgical group—AGB alone, not reported) at baseline [74].

In addition to the small number of nationwide AGB studies with ≥3 years of follow-up, the current outcomes should be compared with those of systematic reviews or meta-analyses, which typically include a wide sample of studies, possibly limiting bias toward COE “center effects.” In considering AGB studies prior to 2003, a systematic review by Chapman et al. concluded that the quality of AGB studies with intermediate follow-up (defined as 2–4 years) was only moderate, and long-term effectiveness (>4 years), unproven [77]. Five years later, a systematic review by Tice et al. (sponsored by US insurer Blue Shield of California comparing 1966–2007 AGB and Roux-en-Y gastric bypass outcomes) found similarly that, despite being a popular procedure, AGB results beyond a single postoperative year were seldom reported [78]. Four other systematic reviews and/or meta-analyses published in the last decade reviewed %EWL and/or BMI change at ≥1 year, though these echo conclusions by Chapman et al. and Tice et al. that ≥3-year AGB outcomes were few, and limited in most studies, by marked patient loss to follow-up [44, 45, 79, 80].

In a 2004 meta-analysis by Buchwald et al. that included 1,848 patients with AGB %EWL data (1995–2003), EWL of 47.5 % at >2 years was nearly identical to that of the current French SAGB study EWL, 47.4 % at 3 years [44]. A 2006 systematic review by O’Brien found 54.8 % weighted mean EWL at 3-year follow-up (AGB *n* = 3,104, 12 studies) [79]; a 2008 meta-analysis by Cunneen et al. comparing SAGB (*n* = 4,274, 33 studies) and Lap-Band (*n* = 24,707, 104 studies) found respective 3-year EWL of 56.4 % and 50.2 % [80]; and a 2009 meta-analysis by Garb et al. comparing AGB and gastric bypass found 55.2 % EWL in the AGB group (*n* = 7,383, 18 studies) at 3 years [45]. While 3-year mean %EWL in the recent three reviews was greater than that of the current nationwide study, as would be expected with a greater proportion of higher-volume sites and inclusive of RCTs, the mean %EWL 95 % confidence interval (95 % CI: 44.0, 51.0) of the current study was found to overlap with 95 % CIs reported in the two most recent and largest meta-analyses, suggestive of relatively comparable outcomes.

Most intermediate-term AGB studies demonstrate high rates of patient attrition, up to 86.2 % at >3-year follow-up [45], and incomplete data collection. Although the current study satisfied the <20 % loss to follow-up with respect to weight and safety data, it was somewhat limited by issues related to incomplete and missing data, particularly in the areas of comorbidity and QoL data collection. Indeed, due to a likely underreporting bias, a reliable quantitative estimate of change in prevalence of any specific comorbidity could not be calculated. Missing QoL data was evident even at baseline, ranging from 13.0 to 42.0 %, again, depending on the complexity of the instrument. In addition, although 91.1 % of patients in this cohort satisfied eligibility criteria, in some cases, eligibility requirements were varied according to individual investigator discretion: Under the protocol requirement of consecutive recruitment, 29 patients (5.6 %) presented with a BMI <35 who either had a severe concomitant comorbidity or maximum adult BMI of ≥45 and were implanted with a SAGB as a re-intervention for a failed or previously complicated bariatric procedure. In the subset analysis of re-intervention patients, it was shown that the inclusion of these patients in the overall cohort calculations had a nonsignificant effect on mean BMI change; indeed, no significant difference in mean BMI was observed at 3-year follow-up between re-intervention patients and SAGB first-intervention patients (32.81 ± 5.6 vs 32.13 ± 5.8, *p* = 0.51).

As longer-term AGB data accumulate and bariatric surgeons and insurers seek to analyze the “big picture” of bariatric surgery, specifically, the clinical effectiveness of the AGB procedure, the opportunity to review published findings from the full range of sites performing bariatric surgery will be important. This report presents the first government-commissioned clinical trial results for the Swedish Adjustable Gastric Band in routine use in multiple centers across France.

SAGB treatment was safe and effective. At 3-year follow-up, 87.0 % of SAGB implants were fully functional and a significant BMI reduction of 9.0 kg/m^2^ with a corresponding 47.4 % EWL was observed. The cohort’s EQ-5D QoL-related utility score improved significantly and was found to be essentially equivalent to that of patients with normal BMI. These findings corroborate prior French countrywide AGB insurance surveys and compare favorably with other national AGB outcome studies, systematic reviews, and meta-analyses. More long-term nationwide studies of AGB outcomes are needed to further clarify reimbursement criteria and refine treatment standards.
